# Hepatobiliary Metastasis of Colorectal Cancer in a Frail Patient and the Role of Immunohistochemistry and Endoscopic Retrograde Cholangiopancreatography (ERCP): A Case Report

**DOI:** 10.7759/cureus.28979

**Published:** 2022-09-09

**Authors:** Mansoor Zafar, Irfan Ullah, Noor Inayat, Hugh Godfrey, Jaber Gasem

**Affiliations:** 1 Gastroenterology, Hepatobiliary, and Hepatology, Royal Sussex County Hospital, University Hospitals Sussex NHS Foundation Trust, Brighton, GBR; 2 Medicine, Nevill Hall Hospital, Abergavenny, GBR; 3 Medicine, District Headquarter Hospital Kohat Development Authority (KDA), Kohat, PAK; 4 Interventional Radiology, Ysbyty Gwynedd Hospital, Bangor, GBR; 5 Gastroenterology, Ysbyty Gwynedd Hospital, Bangor, GBR

**Keywords:** endoscopic retrograde cholangiopancreatogram (ercp), immunohistochemistry staining, percutaneous transhepatic cholangiogram, bile duct metastasis from primary colorectal cancer, cholangiocarcinoma

## Abstract

Primary cholangiocarcinoma (malignancy of the bile ducts) is potentially a treatable malignancy via surgery. It presents with a derangement in the liver function blood test results, which results in raised bilirubin. It may also be accompanied by the complaint of itching of skin, dark urine, and light color stool. Bile duct metastasis from primary colorectal cancer, although a very rare condition presents with similar symptomatology and blood test results. Immunohistochemistry staining of tissue biopsy has significantly improved differentiation, detection, and hence plan management of both malignancies (cholangiocarcinoma and bile duct metastasis from primary colorectal cancer). The role of endoscopic retrograde cholangiopancreatography (ERCP) is important with the insertion of a stent towards symptom relief when palliative management is indicated in patients with an incurable disease.

## Introduction

Colorectal cancers also commonly known as colorectal adenocarcinomas arise from the glandular epithelial layers and undergo mutations [1] transforming them into either adenoma which are of benign nature or carcinomas with a tendency to metastasize over a prolonged period [2]. It has been reported to be the third leading cause of cancer death throughout the world and its reported incidence appears to be rising steadily in developing nations [3]. According to GLOBOCAN 2018 data, the incidence of colon cancer is the fourth, most common worldwide and the incidence of rectal cancer is the eighth most common worldwide. However, together the reported incidence is 10% of all cancer diagnoses and hence is the third most common cancer diagnosed across the world [3-5].

## Case presentation

A 69-year-old male was admitted via the emergency department to the gastroenterology and hepatobiliary ward at a district general hospital with concerns about jaundice and itching of the skin. He had a past history of stable gout, alcohol excess, and Dukes’ B adenocarcinoma of the sigmoid colon. Five years ago, Dukes’ B adenocarcinoma was managed with anterior abdominal resection with ileostomy (which was successfully reversed two years later). Three years ago, he was found to have raised serum carcinoembryonic antigen (CEA) of 13 ug/L. This prompted a computed tomogram (CT) of the abdomen and pelvis which only showed mild fatty infiltration. A request for a magnetic resonance cholangiogram (MRCP) showed the presence of four liver metastases with portocaval node involvement. The patient underwent extended right hemihepatectomy with cholecystectomy and the biopsy confirmed metastatic adenocarcinoma of bowel origin. However, the operation resulted in the complication of the biliary leak, managed with an external drain. His case was discussed in a multidisciplinary meeting (MDM) for consideration for adjuvant chemotherapy or localized treatment with stereotactic ablative body radiotherapy (SABR) for query oligometastatic disease. Initially, chemotherapy was deferred by the oncology team as the patient was 12 weeks post-surgery with continuous bile drainage via drain and with performance status (WHO performance status) of 2. Over the next four weeks, the biliary leak resolved and the patient was discharged home with a performance status of 0. Post-discharge he underwent portocaval SABR 30 gray (Gy) in 5 fractions (#5) with six monthly periodic reviews with surveillance imaging in the hepatobiliary clinic as an out-patient with no concerns.

**Figure 1:**
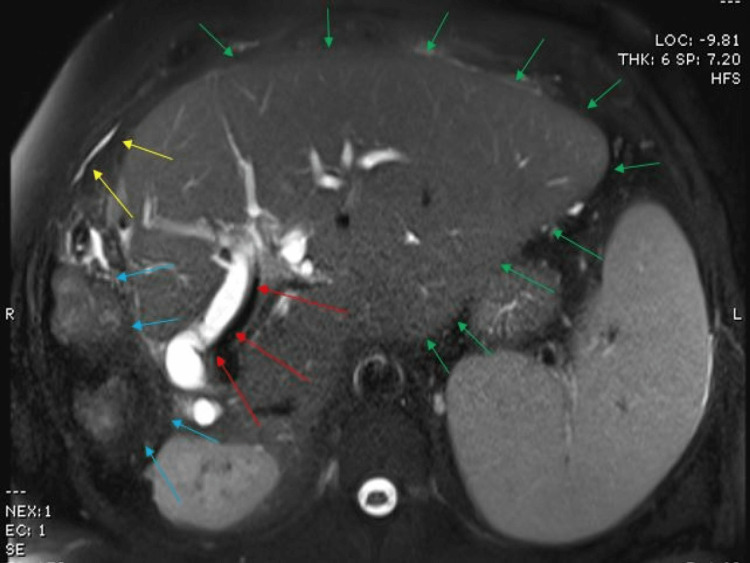
MRCP shows post-hepatectomy with resection of segments 5, 6, and 7 (blue arrows), dilated common bile duct (CBD; red arrows), and significant hypertrophy of left lobe of liver (green arrows). Mild sub-phrenic abscess (yellow arrows) is noted. MRCP: magnetic resonance cholangiogram

**Figure 2:**
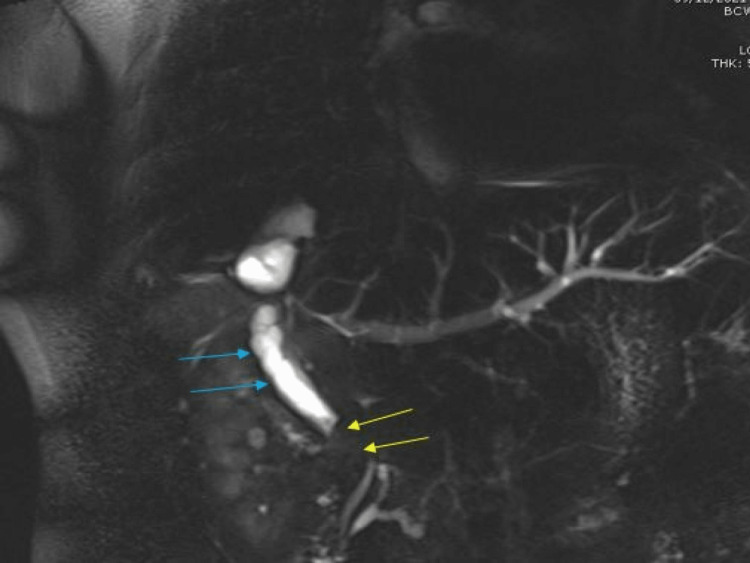
MRCP shows dilated common bile duct (CBD) with filling defect (yellow arrows) and no calculus concerning biliary stricture (blue arrows). MRCP: magnetic resonance cholangiogram

He was followed in hepatobiliary clinic and two years after partial hepatectomy, he was noticed to be icteric and pruritic with mild pain in the right upper quadrant of the abdomen with no guarding and no rebound tenderness. His medications included colchicine, allopurinol, and lansoprazole. His CT abdomen showed a small collection superiorly along the previously resected liver margins and some prominent lymph nodes with no clear evidence of metastatic recurrence. An urgent MRCP was requested to rule out any recurrence of metastatic cancer at the hepatic anastomotic site. On further inquiry, the patient mentioned he had been feeling unwell for the last two weeks with nausea, decreased appetite, and itching of his skin with dark color urine and clay-colored stools. He admitted to having been drinking alcohol about 2 pints a day and agreed to cut down on his alcohol. He was advised for admission but he declined at that time as he felt safer at home with support from relatives and friends. He agreed to have complete liver screening and blood tests to assess derangement in his liver function. The blood tests reflected completely normal liver screen and serum paracetamol levels albeit, deranged liver function tests (LFTs) with an obstructed picture. MRCP reported dilated common bile duct (CBD) with no evidence of calculus with a concern for CBD stricture with hypertrophy of the remaining left lobe of the liver and small sub-phrenic abscess along the previously resected segments along the right lobe of the liver (Figures 1, 2).

**Figure 3:**
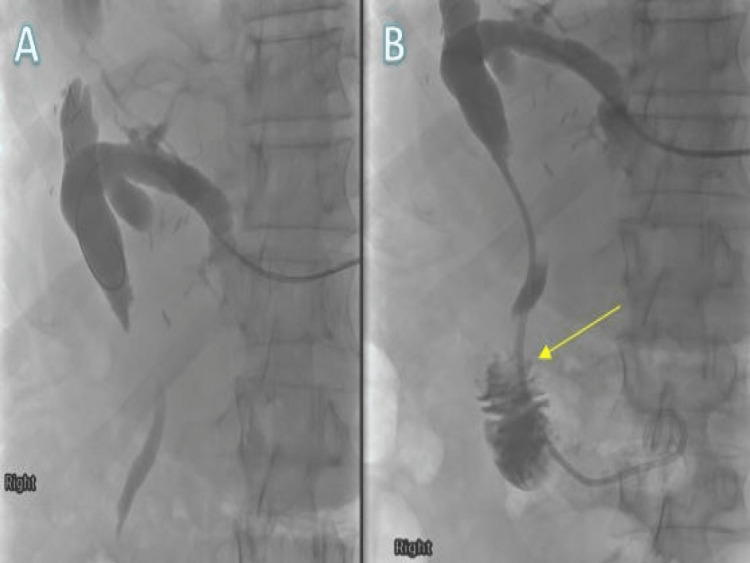
PTC prior to entering ampulla (A) after percutaneous internal-external transhepatic biliary drainage pass duodenal ampulla (B) (yellow arrow). Catheter placement is successful in obtaining the tissue sample for biopsy. PTC: percutaneous-transhepatic cholangiogram

His case was discussed in a multidisciplinary meeting (MDM), and a decision was made to have a percutaneous-transhepatic cholangiogram (PTC) via the left lobe of the liver, with an aim to collect brushing or biopsies for tissue diagnosis and an attempt to decompress the CBD. The patient was admitted for two days, and approximately 2 litres of bile drain via an external drain was administered while he was on broad-spectrum intravenous antibiotics (Figures 3A, 3B).

**Figure 4:**
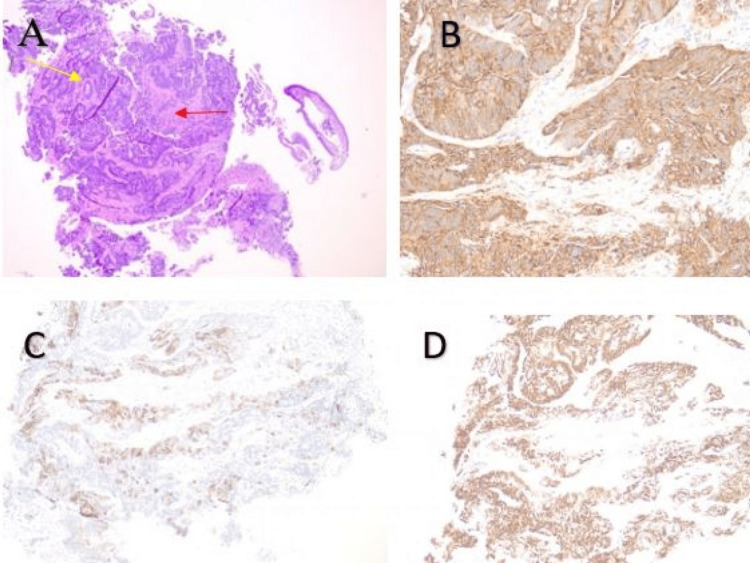
Histopathology from tissue sample obtained at the time of PTC. The images show (A) H&E ×5 with metastatic cancer cells of colorectal origin (red arrow) interspersed along the normal lining (yellow arrows), (B) immunohistochemical caudal-type homeobox 2 (CDX2) stain, (C) cytokeratin 2 (CK2) stain, and (D) special AT-rich sequence-binding protein 2 (SATB2) stain. PTC: percutaneous-transhepatic cholangiogram

**Figure 5:**
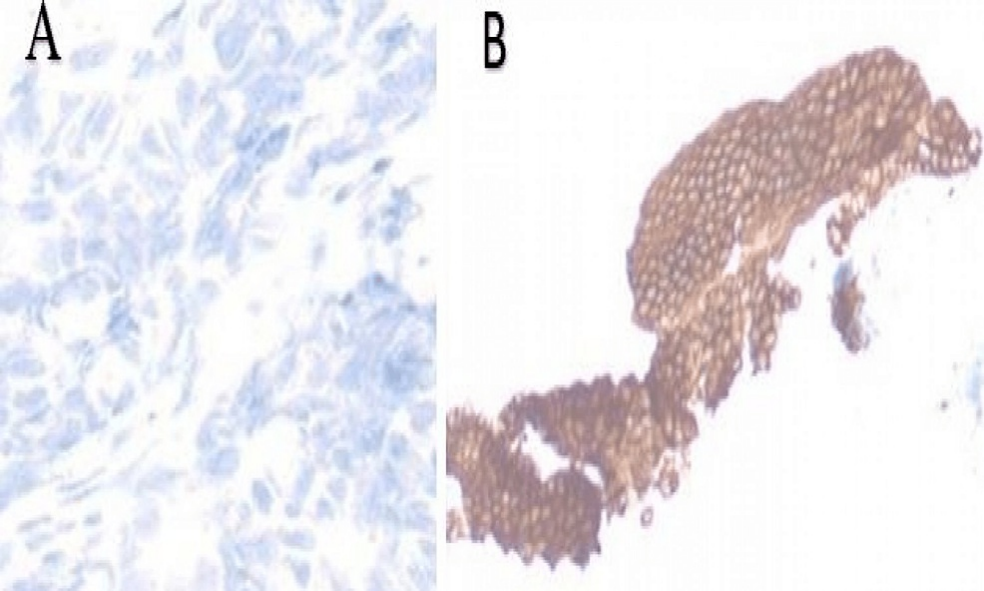
Histopathology from tissue sample obtained at time of PTC. The images show (A) CK7 negative stain and (B) cytokeratin (CK) positive stain in background of biliary epithelium. PTC: percutaneous-transhepatic cholangiogram

However, the external drain accidentally came out and a temporary stoma was placed to collect the residual drain. The team decided to wait for biopsy results while the patient managed conservatively with intravenous broad-spectrum antibiotics and intravenous fluids as an in-patient. The biopsy confirmed metastatic adenocarcinoma of colorectal origin (Figures 4A-4D and Figures 5A, 5B).

**Figure 6:**
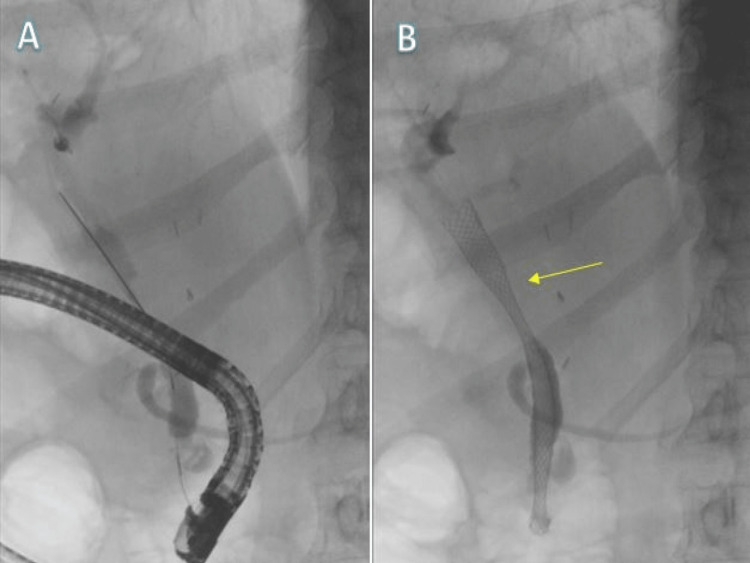
ERCP (A) before stent insertion and (B) after successful stent insertion (yellow arrow). ERCP: endoscopic retrograde cholangiopancreatography

His case was again discussed in MDM and a decision was made to have a stent insertion to decompress the biliary duct, with the patient's consent. Following this, a 6-cm fully covered metal stent was inserted during endoscopic retrograde cholangiopancreatography (ERCP) in the CBD (Figures 6A, 6B).

Following that he was given vitamin K 10 mg intravenous for three days for raised international normalized ratio (INR) of 6.9. This resulted in the normalization of INR, improvement in LFTs including serum bilirubin levels, and resolution of the symptoms of jaundice and pruritus* *(Table 1).

**Table 1:** Blood test results and improvement in serum markers.

Lab parameter tested	Units	Reference	Day 1	Day 5	Day 7
Hemoglobin (Hb)	g/L	130-180	139	137	137
Mean corpuscular volume (MCV)	fL	80-100	96	94	95
Platelet	x10^9^/L	150-400	227	223	223
White blood cells (WCC)	x10^9^/L	4-11	8.9	6.5	4.0
Neutrophils	x10^9^/L	1.5-8	54	43	-
Albumin	g/L	35-50	6.2	4.7	2.9
Bilirubin	umol/L	0-21	69	24	28
Alkaline phosphatase (ALP)	U/L	30-130	452	398	328
Alanine transaminase (ALT)	U/L	10-35	124	94	81
Gamma glutamyl transaminase (GGT)	U/L	0-30	628	-	-
International normalized ratio (INR)	-	0.8-1.2	6.9	-	1.2
Ferritin	μg/L	41-400	1014	-	-
Iron	μmol/L	14-32	5	-	-
Transferrin saturation	%	20-50	14.2	-	-

Following three days post-procedure, the patient was discharged and switched to oral linezolid for 14 days with follow-up care by the oncology team to review. He also had a staging CT chest, flexible sigmoidoscopy, and CT colonoscopy to rule out any recurrence of colorectal cancer during this admission, and the findings were nil acute. One year post-ERCP with a stent, aged 70 years, during hepatobiliary clinic review, he remains mobile with frame with Rockwood (Dalhousie University frailty score) frailty score of 5. His MRI confirmed a new lesion less than 1 centimeter (cm) in segment 3 of the liver and positron emission tomogram-computed tomogram (PET-CT) confirmed increased uptake adjacent to the biliary stent. He remains under the care of the palliative team and had one cycle of oxaliplatin and capecitabine which he tolerated well and remains under periodic review with hepatobiliary and palliative teams as an out-patient for an incurable disease.

## Discussion

The global burden of colorectal cancer is predicted to rise to over 2.2 million new cases (an increase of 60%) and annual deaths to rise to 1.1 million by the year 2030 [6]. This has been reported to be associated with the use of processed food, alcohol use, meat consumption, a sedentary lifestyle, an increase in body-mass index (BMI), and greater longevity [6]. Edwards et al. have credited polypectomy, screening colonoscopies, flexible sigmoidoscopies, computed tomography (CT) colonography, fecal immunochemistry, and fecal occult blood testing with better survival from colorectal cancer [7]. Although the incidence rate for colorectal cancer has decreased over the past decades for ages 50 years or more, the incidence rate has increased for those age under 50 years of age suggesting recommendations for the screening age to be 45 years instead of 50 years [8].

Among men with no recurrence of colon cancer five years after the previous diagnosis, one in 12 patients has been reported to have recurrence between five and 10 years time, with a cumulative rate of 7.8%. For women, the frequency was one in 19 with a cumulative rate of 5.2%. However, in the multivariate analysis, female sex and age under 75 years have been reported to be associated with a lower risk of recurrence. The stage at diagnosis has not been a predictor of late recurrence. Late recurrence following the colon cancer resection with curative intent has been reported earlier [9].

First reported by Herbut and Watson [8] in 1946, biliary metastasis from colon cancer remains a very rare manifestation [8,10,11]. It has been argued that the isolated biliary metastasis is a less aggressive variant of hepatic metastasis with a possibly better prognosis than a hepatic parenchymal metastasis from colorectal cancer [12]. Jennings et al. have described two variants of biliary metastasis which could either present as a malignant biliary stricture (as in our patient on CT scan) or as an endoluminal lesion [13].

The biliary dilatation following imaging to rule out obstructive causes often raises concerns about the possibility of cholangiocarcinoma [13]. However, the immunohistochemical staining with a positive result for cluster of differentiation 20 (CD20) and negative staining for CD7 has enormously helped to pinpoint a possible intestinal origin as in our patient [14,15]. Primary cholangiocarcinoma has been reported to express cytokeratin 7 (CK7) [16]. Colorectal cancers on the contrary have been reported to express CK20 [16]. Koh et al. have reported case series of five patients who either had primary malignancy located in the sigmoid colon (n=3, 60.0%) or the ascending colon (n=2, 40.0%) [17]. They have reported a median time from the diagnosis of colorectal cancer to biliary metastasis of 59.2 months (0-70.1 months) [17]. The primary tumor had been resected in three patients (60%) and they had developed extra-colonic metastasis prior to the occurrence of biliary metastasis. The biliary and intrahepatic spread was seen in two patients (40%) and only one patient (20%) had dissemination to the biliary, lung, and liver involvement [17]. The importance of immunohistochemistry stems from the fact that primary cholangiocarcinoma is potentially surgically treatable. These reported surgical options include hemihepatectomy with resection of the extra-hepatic bile duct, trisectionectomy extended to second‐order biliary radicals, reconstruction of multiple right‐sided ducts, or pancreatoduodenectomy [18].

We report a patient who had a prior diagnosis of sigmoid cancer managed surgically, followed by hepatic involvement managed with surgical resection. The patient then presented with deranged LFTs with obstructive pathology five years later, when he was initially queried for cholangiocarcinoma. However, the tissue sample followed by immunohistochemical staining helped to diagnose the recurrence of metastatic colorectal condition five years after the initial surgery. It also highlights the importance of ERCP with stent insertion as a means of symptom relief in a patient with an incurable disease.

## Conclusions

Biliary metastasis although very rare, remains an important differential in a patient presenting with painless jaundice, with a previous history of colorectal cancer. Immunohistochemical tests are vital to differentiate a primary biliary malignancy from the prior colorectal origin.

Although biliary metastasis has a poor outcome there are palliative treatment options via ERCP stent insertion for symptom relief. A regular follow-up in the clinic at six-month intervals (surveillance) is necessary to detect early signs of possible recurrence of metastatic disease.
